# Long-term potentiation at pyramidal cell to somatostatin interneuron synapses controls hippocampal network plasticity and memory

**DOI:** 10.1016/j.isci.2022.104458

**Published:** 2022-05-27

**Authors:** Azam Asgarihafshejani, Ève Honoré, François-Xavier Michon, Isabel Laplante, Jean-Claude Lacaille

## Main Text

(iScience *25*, 104259; May 20, 2022)

In the originally published version of this article, the illustration in Figure 2A mistakenly included a stimulation electrode, which should not be present in this schematic. This error has now been corrected in the article online. The authors apologize for any confusion this error may have caused.

